# pRb controls Estrogen Receptor alpha protein stability and activity

**DOI:** 10.18632/oncotarget.1036

**Published:** 2013-06-03

**Authors:** Isabella Caligiuri, Giuseppe Toffoli, Antonio Giordano, Flavio Rizzolio

**Affiliations:** ^1^ Sbarro Institute for Cancer Research and Molecular Medicine, Center for Biotechnology, College of Science and Technology, Temple University, Philadelphia, PA, USA; ^2^ Department of Medicine, Surgery and Neuroscience, University of Siena, Siena Italy; ^3^ Division of Experimental and Clinical Pharmacology, Department of Molecular Biology and Translational Research, National Cancer Institute and Center for Molecular Biomedicine, Aviano (PN)

**Keywords:** pRb, Estrogen receptor alpha, proteasome, chaperone proteins, breast cancer

## Abstract

A cross talk between the Estrogen Receptor (ESR1) and the Retinoblastoma (pRb) pathway has been demonstrated to influence the therapeutic response of breast cancer patients but the full mechanism remains poorly understood. Here we show that the N-terminal domain of pRb interacts with the CD domain of ESR1 to allow for the assembly of intermediate complex chaperone proteins HSP90 and p23. We demonstrated that a loss of pRb in human/mouse breast cells decreases the expression of the ESR1 protein through the proteasome pathway. Our work reveals a novel regulatory mechanism of ESR1 basal turnover and activity and an unanticipated relationship with the pRb tumor suppressor.

## INTRODUCTION

The histological and molecular classification of breast cancer (BC) has drastically helped to characterize and treat patients, although the clinical resolution is an enigma. A limited number of biomarkers can be used to manage BC patients and the estrogen receptor alpha (ESR1) is essential for hormonal therapy. Unfortunately, almost 30% of BCs are ESR1 negative or acquire resistance to hormonal therapy [1,2]. Among specific biomarkers that predict the response to therapy or can be utilized as new therapeutic targets, pRb is a strong candidate [2].

The pRb pathway is frequently inactivated in breast cancer via a phosphorylation dependent mechanism that is driven by the overexpression of cyclin D1 or inactivation of the CDK-inhibitor p16ink4a. pRb gene or protein loss has also been reported at different frequencies [3]. Deletion of Rb1 in mouse mammary stem/bipotent progenitor cells induced focal acinar hyperplasia with squamous metaplasia that progressed in transplantable mammary tumors similar to either luminal-B or TNT subtypes [4].

pRb is a multifaceted tumor suppressor protein that controls many pathways but has been well-described only in its role in cell cycle control. pRb acts as a repressor of the cell cycle by inhibiting the activity of E2F transcription factors. Hyper-phosphorylated pRb releases E2F transcription factors and allows for the expression of genes that mediate entry into the S phase [5]. Emerging evidence suggests that pRb has a more complex role in cancer initiation and progression [6] and understanding which functional biological nodes are altered in pRb negative cells is an important question in order to realize personalized therapy.

There is a convincing association between ESR1 and pRb status. Histological analyses of different breast cancer subtypes showed a prevalence of pRb loss in ESR1 negative tumors [7] with a high frequency of occurrence in triple-negative breast cancer subtypes [8]. From a therapeutic point of view, a gene expression signature of pRb-dysfunction is associated with a relatively poor response to endocrine therapy and a better prognosis following chemotherapy treatment that is widely utilized in the treatment of ER-negative disease [3,8–10].

These data suggest an interaction between the pRb pathway and the status of ESR1. In turn, there is some evidence in the literature that has driven us to hypothesize a direct link between the pRb and ESR1 protein functionality. pRb is a cofactor for more than a hundred proteins [6] including nuclear receptors. Rb indirectly enhances the activity of glucocorticoid receptors and inhibits the thyroid hormone receptor and PPARgamma-dependent transcription [11]. In cancer cells, pRb modulates the activity of the AR and ESR1, the two principal determinants of hormonal cancer. pRb interacts with the androgen receptor in a hormone-independent manner (Lu and Danielsen jbc 1998) [12] and can regulate its activity via the E2F transcription factor 1 resulting in a critical determinant of therapeutic response [13]. Finally, pRb interacts in a ligand-dependent manner with the RIZ protein, an ESR1 cofactor that can also potentiate SRC-2 activity on ESR1 signaling [11]. Conversely, the direct activity of pRb on ESR1 protein function remains largely unknown.

Here we show that the retinoblastoma protein (pRb) is fundamental for ESR1 basal turnover and activity. We demonstrated that loss of pRb in human breast cancer cells or human/mouse primary mammary cells, but not the two related family members p107 and pRb2/p130, decreases the expression of the ESR1 protein. Treatment with proteasome inhibitors re-establishes the expression of ESR1 demonstrating the involvement of the proteasome pathway. As confirmed, in *RB1* knock down cells, ESR1 is ubiquitinated to a greater degree than in normal cells. Mechanistically, the N-terminal domain of pRb interacts with the CD domain of ESR1 to allow for the interaction of chaperone proteins and in particular, HSP90 and p23. We demonstrated that pRb is important for the formation of a chaperone intermediate complex on ESR1.

## RESULTS

### pRb controls the ESR1 protein level and activity

**Figure 1:**
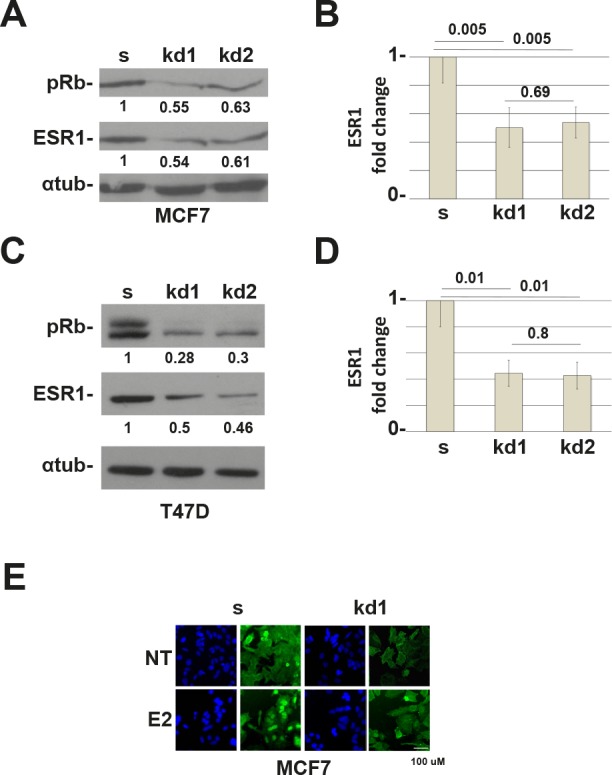
RB1 kd MCF7 and T47D cells down regulate the ESR1 protein (A) ESR1 expression in *RB1* knocked down MCF7 cells. kd1 and kd2 represent two different shRNAs. Alpha-tubulin was used as a loading control. (B) Quantification of three independent experiments as in (A). y axis represents the ratio between the ESR1 and alpha-tubulin proteins and normalized to scrambled cells. The *RB1* knocked down MCF7 cells express about 50% less of the ESR1 protein. (C) ESR1 expression in *RB1* knock down T47D cells. (D) Quantification of three independent experiments as in (b). The *RB1* knock down T47D cells express about 56% less of the ESR1 protein. (E) MCF7 cells were grown in CSS medium for 3 days (NT) and treated with 10^−8^ estradiol for 45 minutes (E2). Cells were probed with an ESR1 antibody (green) and stained with DAPI (blue). *RB1* kd1 cells express less ESR1 protein.

**Figure 2:**
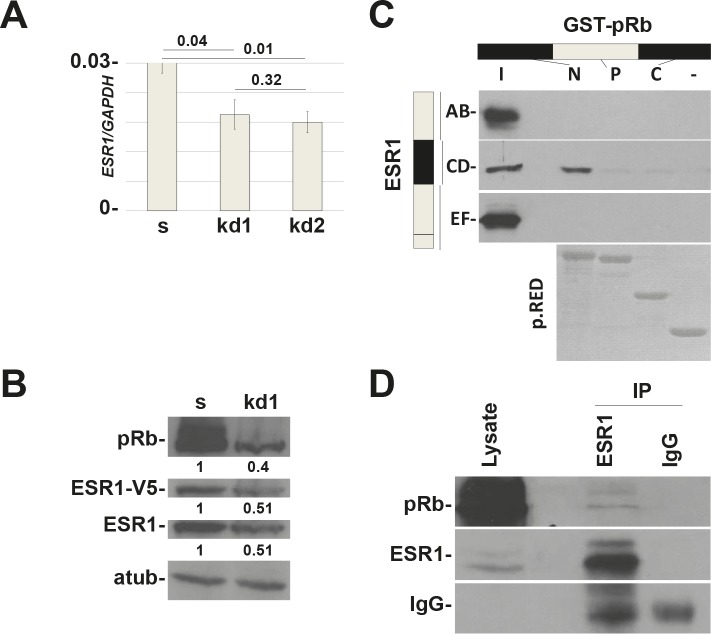
In vitro and in vivo interaction between pRb and ESR1 proteins (A) *RB1* kd1 and kd2 MCF7 cell lines have about 30% less mRNA levels of ESR1. y axis represents the ratio between *ESR1* and *GAPDH* mRNAs. (B) MCF7 cells were infected with a lentivirus that expresses ESR1-V5 under the CMV promoter. *RB1* kd1 cells express less endogenous and transfected ESR1 protein, demonstrating that the effect is at the posttranslational level. Quantification represents the ratio between the indicated protein and alpha-tubulin and normalized to scrambled cells. (C) *In vitro* interaction. ESR1 AB, CD and EF domains were pulleddown with GST-pRb N-terminal, Pocket and C-terminal domains in MCF7 cells. There is an interaction between the ESR1CD and pRb N-terminal domains. p.RED, pounceau red. (D) *In vivo* interaction. ESR1 was immunoprecipitated in MCF7 cells and analyzed by western blot with a pRb antibody. IgG was used as a negative control.

To test our hypothesis, we have generated MCF7 (ESR1 positive) cell lines knocked-down for the three members of the Retinoblastoma family, pRb, pRb2/p130, and p107 (Supplemental Figure 1A) [14,15]. Supplemental Figure 1B shows that the loss of pRb family members decreased the expression of ESR1 when compared to scrambled cells. Among the three members, only pRb is involved in this mechanism (Supplemental Figure 1B, Figure 1A). The data were obtained in basal conditions in the absence of hormones (Charcoal Stripped Serum, CSS). We decided to perform all the experiments under these conditions unless otherwise indicated. To exclude that the mechanism is a characteristic of a single cell line, we have down regulated pRb in the T47D ESR1 positive breast cancer cell line. The results in T47D cells are comparable to those in the MCF7 cells (Figure 1C). In both cell lines, the downregulation of ESR1 in *RB1* kd cells is statistically significant (Figure 1B,D). To confirm the data, we have carried out immunofluorescence experiments. We observed a reduction in signal intensity of the ESR1 in MCF7 *RB1* kd cells in basal and estradiol-stimulated conditions (Figure 1E). To definitively demonstrate that the activity of ESR1 was compromised, we have assessed the expression of some classical ESR1 target genes [16]. We observed that the expression of *TFF1* and *CTSD* are down regulated in *RB1* kd cells (Supplemental Figure 1C). Analysis of ESR1 mRNA also showed a reduction in *RB1* kd cells (Figure 2A). Since the pRb family members could bind the ESR1 promoter [17] and the ESR1 protein itself regulates its expression [16], we have cloned the ESR1 downstream a non-endogenous promoter. A western blot analysis indicates that the reduction of ESR1 relative expression under the non-endogenous promoter is comparable with that of endogenous promoter indicating a control at the posttranscriptional level (Figure 2B). These data indicate that pRb could be a new cofactor of ESR1, regulating its protein expression level.

### pRb and ESR1 form a protein complex

To test a possible interaction between pRb and ESR1, we have carried out a GST pull down assay with the three functional domains of pRb [5,18] and the AB, CD and EF domains of the ESR1 protein in MCF7 cells [19]. As highlighted in Figure 2C, the pRb N-terminal domain interacts with the CD domain of ESR1. To definitively demonstrate this interaction, we have performed a co-immunoprecipitation assay on endogenous proteins. Figure 2D shows the *in vivo* interaction between pRb and ESR1.

### pRb controls the basal turnover of ESR1 via the proteasome pathway

**Figure 3:**
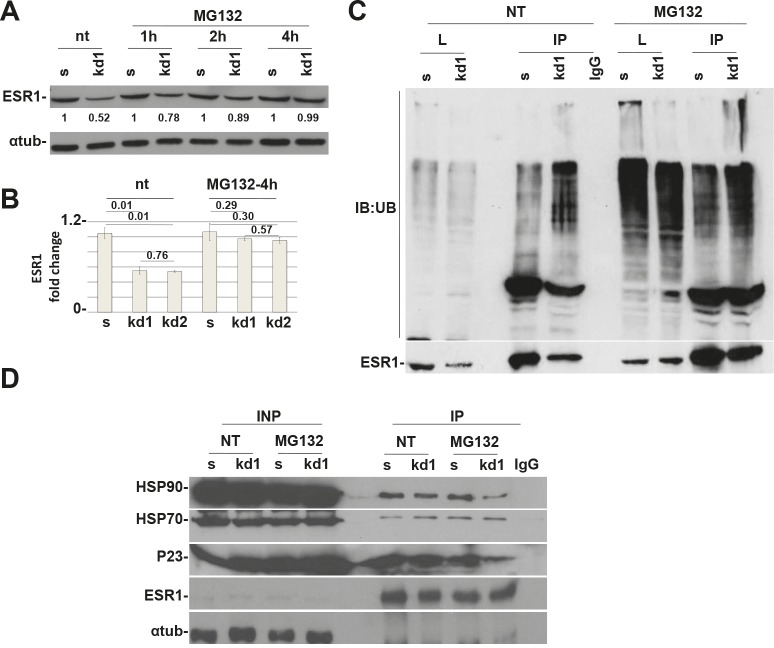
pRb is necessary for HSP90 protein to protect ESR1 from proteasomal degradation (A) Time course analysis of MCF7 cells treated with MG132 for indicated time. After 4 hours, the *RB1* kd1 cells expressed the same level of ESR1 as scrambled cells. (B) Analysis as in (A) of three independent experiments utilizing *RB1* kd1 and kd2 shRNAs. (C) MCF7 cell lysate untreated (nt) or treated with MG132 for 4 hours was immunoprecipitated with an ESR1 antibody and analyzed by western blot with an anti-ubiquitin antibody. The *RB1* kd1 cells showed heavy ubiquitination that extended in the stacking gel. (D) MCF7 cell lysate untreated (nt) or treated with MG132 for 4 hours was immunoprecipitated with ESR1 antibody. In *RB1* kd1 cells, there is less HSP90 and p23 proteins bound to the ESR1 protein. HSP70 protein is unaltered. IgG was used as a negative control.

**Figure 4:**
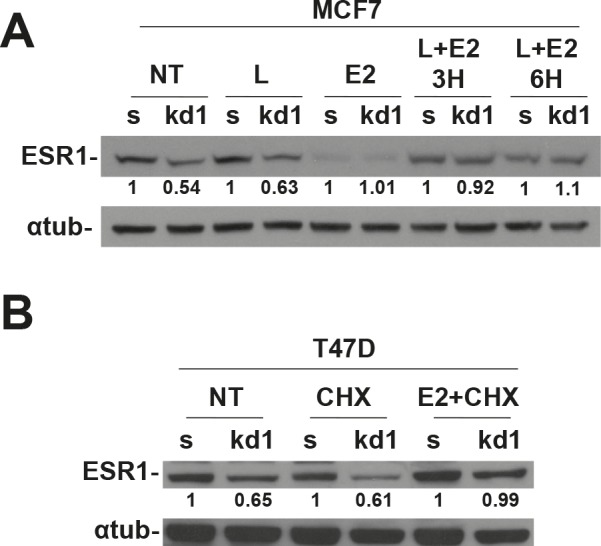
Nuclear accumulation of ESR1 prevents proteasomal degradation in RB1 kd1 cells (A) MCF7 cells were treated with leptomycin B, estradiol or a combination for indicated time. Estradiol allows the translocation of ESR1 in the nucleus and Leptomycin B prevents the nuclear export. After 3 or 6 hours of treatment, the *RB1* kd1 cells express the same level of ESR1 as scrambled cells. (B) T47D cells were treated with CHX, estradiol, or a combination for 3 hours. CHX allows nuclear accumulation of ESR1. After CHX plus estradiol treatment, the *RB1* kd1 cells express the same level of ESR1 as scrambled cells.

ESR1, as with most of the hormonal receptors, is finely regulated at the transcriptional and posttranscriptional levels. A key role in protein half-life is played by the proteasome pathway [19–21]. Without the hormone, the ESR1 is associated with HSP70, HSP40 and the adapter HIP protein (HSP70-interacting protein) to form an early complex. Later on, HSP90 and the adapter protein HOP (HSP70/HSP90- organizing protein) displace HSP40 and bind the hydrophobic hormone-binding domain of ESR1 to form an intermediate complex [22]. After ATP binding, HSP90 interacts with p23 and Cyclophilin 40 (CYP40) to form a mature complex [22]. To test if pRb is involved in the ESR1 degradation via the proteasome pathway, we have treated the MCF7 cells with the proteasome inhibitor MG132 [23]. After 4 hours of treatment, *RB1* kd cells had the same level of ESR1 as scrambled cells, thus rescuing the phenotype observed in untreated cells (Figure 3A,B). This finding was confirmed in *RB1* kd2 cells (Supplemental Figure 2A) and utilizing the drug Bortezomib (Supplemental Figure 2B), another proteasome inhibitor. The same results were also observed in T47D cells (Supplemental Figure 2C). To assess if ESR1 is more ubiquitinated in *RB1* kd MCF7 cells, we treated the sample with MG132, immunoprecipitated with an ESR1 antibody and analyzed the ubiquitin level. Figure 3C confirmed that ESR1 is more ubiquitinated in *RB1* kd cells as compared to scrambled cells. A typical smear is observed in the stacking gel. As a consequence of the obtained results, we have analyzed the chaperone proteins involved in ESR1 protein stability [24]. In Figure 3D, we show the co-immunoprecipitaion of ESR1 with HSP90. It appears that *RB1* kd1 cells have less HSP90 bound to ESR1 in untreated and MG132-treated samples compared to scrambled cells. The HSP90 co-chaperone, p23, is also reduced as expected. The level of HSP70 is unaltered, suggesting that pRb influences the intermediate complex that stabilizes the ESR1 protein [22]. Under basal conditions, the ESR1 is associated with chaperone proteins in the cytoplasm. After estradiol stimulation, ESR1 is shuttled to the nucleus to exert its genomic function on target genes [25]. The re-cycling of ESR1 on promoters of target genes is very fast [26], after which the ESR1 is exported again to the cytoplasm for proteasomal degradation [23]. Inhibitors of nuclear export such as Leptomycin B [27] or, nuclear stabilizing agents such as cycloheximide lead to ESR1 protein accumulation. When *RB1* kd1 cells were treated with Leptomycin B (Figure 4A) or cycloheximide (Figure 4B) in combination with estradiol, the level of ESR1 were comparable with scrambled cells, confirming a cytoplasmic ESR1 degradation driven by the proteasome pathway.

### pRb controls ESR1 protein levels in primary human mammary cells and Rb1 KO mice

**Figure 5:**
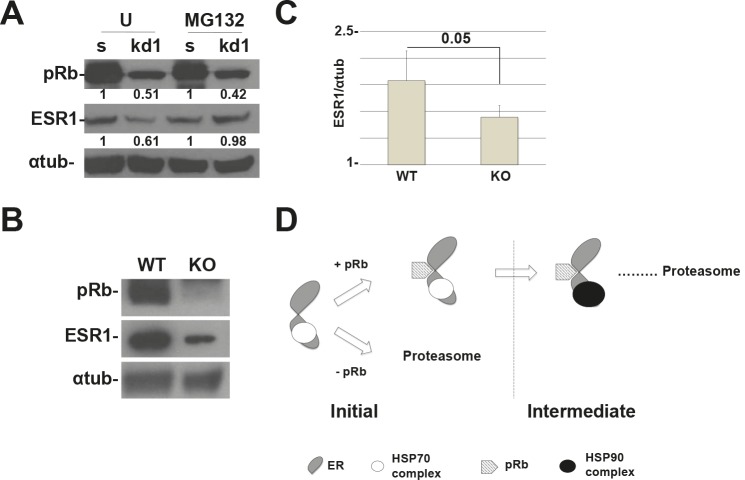
RB1 kd primary human mammary cells and Rb1 KO mice show a reduction in ESR1 protein levels (A) HMEpC *RB1* kd1 cells express 50% less of the ESR1 protein compared to scrambled cells. Treatment with MG132 for 4 hours rescued the expression of the ESR1 protein. The quantification represents the ratio between the ESR1 and alpha-tubulin proteins and normalized to scrambled cells. (B) *Rb1* conditional KO mice in mammary cells have a reduced expression of ESR1. (C) Quantification of 6 mice as in (B). (D) Working model of pRb and the chaperone complex that regulate ESR1 turnover. After the assembly of the initiation complex, pRb is necessary for the recruitment of the HSP90 complex that forms the intermediate complex. Without pRb, the ESR1 is more ubiquitinated and degraded by proteasome pathway.

To assess if the mechanism takes place in a more physiological model, we have knocked-down *RB1* in human mammary epithelial primary cells (HMEpC). In these cells, we were able to detect the short isoform of ESR1 [28]. In Figure 5A, we show that *RB1* kd cells have a reduction in ESR1 levels when compared to scrambled cells and MG132 can rescue the phenotype as in MCF7 and T47D cancer cell lines. To definitively demonstrate the role of pRb in the control of ESR1 protein levels, we have conditionally knocked out (KO) the *Rb1* gene in the mouse mammary epithelium. Western blot analysis shows that the expression of the pRb protein is reduced by 50% in KO mice when compared to wild type mice (Figure 5B,C).

## DISCUSSION

Our data here show that the pRb protein plays an important role as an adaptor in the regulation of ESR1 stability and functionality, a key player in hormonal therapy. The N-terminal domain of pRb interacts with the ESR1 CD domain, which is composed of the DNA binding domain (C) and the hinge region (D). The hinge region has recently been discovered to have a complex function. This domain contains a nuclear translocation signal and includes estrogen-independent regulatory sequences, which mediate the interaction with c-Jun and Sp-1 transcription factors [29]. In addition, the domain is modified by many posttranslational modifications [30,31] and together with the ligand binding domain form a surface for HSP90 binding which protects unliganded ESR1 from basal turnover [32]. We anticipate that pRb represents a new actor for the CD domain, which is fundamental for the binding of HSP90, thus regulating the fine equilibrium of rapid switch on/off that characterizes the activity of ESR1 protein. It is interesting to note that the N-terminal domain of pRb mediates this interaction. Although most of pRb's interactions were mapped in the pocket domain, recently the N-terminal domain has gained much attention. It is involved in inhibiting the E2F transactivation ability [33], can interact with different proteins that control DNA replication [34], and form a complex with SRC-2, a coactivator of nuclear receptors including ESR1 [11]. There is a growing body of evidence that the N-terminal domain may have a role in tumor suppression [6,35,36] and our work highlights its importance in the regulation of ESR1 activity with a direct consequence in the response of breast cancer therapy.

Finally, previous papers have shown the pRb can act as cofactor to control the stability of different target proteins [6,36–38]. Interactions between pRb and the RIM domain of E2F1 or with PDX-1, a transcription factor involved in pancreas development and adult β-cell functions, protects these proteins from the ubiquitin-proteasome pathway degradation [36–38]. Our model suggests a role for pRb as a cofactor that allows the interaction between HSP90 and ESR1 to form the chaperone intermediate complex. In the absence of pRb, the formation of the intermediate complex does not occur, thus priming the ESR1 for proteasomal degradation (Figure 5D). In accordance with the literature, blocking HSP90 function with geldanamycin disrupts the interaction with the ESR1 and promotes it's degradation [39].

During tumor evolution, most hormonal-dependent cancers lack hormone responsiveness and dependence. Since steroid hormones induce differentiation, one explanation is that uncontrolled tumor proliferation is incompatible with differentiation. In this context, our data suggest the pRb loss, a central player of cellular proliferation and differentiation, dictates the steroid response and tumor growth. Current breast cancer therapy is based on a few molecular targets [40–44] and it has been challenging to find new candidates thus far. pRb loss can define a class of breast tumors that would experience little benefit from endocrine therapy [2,45]. Therapies that aim to reactivate the function of the pRb protein [9] can help those patients that are not responsive to hormonal therapy.

## METHODS

### Cells culture conditions

MCF7 and T47D breast cancer cell lines were purchased from American Type Culture Collection, Rochville, MD, USA, 293FT cell lines were from Invitrogen Carlsbad, CA, USA and HMEpC from Cell Application, San Diego, CA, USA. Cells were grown at 37 °C, in a 5% CO_2_/95% atmosphere according to the manufacturer's guidelines. Hormone-free medium was prepared with phenol red–free DMEM with 2 mmol/L L-glutamine, 0.1 mmol/L nonessential amino acids, 50 units/mL penicillin, 50 μg/mL streptomycin, and 3% charcoal-stripped FBS.

### Reagents

Antibodies for the following proteins: ERα (sc-8002), ERα HC-20 (sc-543), ERα MC-20 (sc-542), pRb (sc-102), Hsp70 (sc-24) and Hsp90 (sc-69703) were purchased from Santa Cruz Biotechnology, Santa Cruz, CA, USA; α-tubulin (T-6074) from Sigma Inc., St Louis, MO, USA; P23 (MA3414) from Thermo Scientific Pierce; and anti-ubiquitin P4DI from Covance, Princeton, NJ, USA. MG-132 (10012628) from Cayman Chemical; Bortezomib (B-1408) and Leptomycin B (L-6100) from LC Laboratories; Cycloheximide (c4859) from Sigma; and (Z)-4-Hydroxytamoxifen from Enzo Life Sciences.

### Plasmids

shRNA plasmids *RB1* (TRCN0000010418, TRCN0000040167) were from Sigma Inc., St Louis, MO, USA. Scrambled shRNA (Addgene: 17920), psPAX2 packaging plasmid (Addgene: 12260), and pMDG.2 envelop plasmid (Addgene: 12259) were from Addgene Inc, Cambridge, MA, USA. ERα- His-AB, His-CD and His-EF plasmids were derived from VP16-ERα (Addgene: 11351) following amplification with primers: AB-BamHI-F ATG GAT CCA CCA TGA CCA TGA CCC TCC-3', AB-EcoRI-Rev 5'-ATG AAT TCT CCT TGG CAG ATT CCA TAG-3', CD-BamHI-F 5'-ATG GAT CCA CCA TGG CCA AGG AGA CTC GCT ACT GTG-3', CD-EcoRI-Rev 5'-ATG AAT TCT TCT TAG AGC GTT TGA TCA TG-3'. EF-BamHI-F 5'-ATG GAT CCA CCA TGT CTA AGA AGA ACA GCC TGG CC-3', EF-EcoRI-Rev 5'-ATG AAT TCC AGA CCG TGG CAG GGA AAC-3'. After BamHI/EcoRI double digestion, fragments were ligated into a pcDNA6 His/Myc vector. To generate the ERα-V5 plasmid, we have recombined the pLOVE vector (Addgene:15948) and the pENTR223- ERα, (DF-HCC: HSCD00376961) utilizing the Gateway Cloning System (Invitrogen). GST-Rb N-terminal (1-373 a.a.), Pocket (379-792a.a.) and C-terminal (793-920 a.a.) were cloned in PGEX-2T. All the plasmids were sequence verified.

### Lentiviral production

To generate knock down cells, lentiviral particles were produced as described (http://www.broadinstitute.org/genome_bio/trc/publicProtocols.html) by Rizzolio *et. al* [5].

### Real-time PCR

Total RNA was prepared from tissues using the RNA extraction kit RNAeasy (Qiagen Inc, Valencia, CA, USA). One μg of total RNA was reverse transcribed in a 20 μl reaction using M-MLV reverse transcriptase (Invitrogen, Carlsbad, CA, USA). Primers to amplify ER, CTDS, TFF1, GAPDH are the following: ESR1-for 5' -CAT TCT ACA GGC CAA ATT CAG - 3', ESR1-rev 5' -GCA CAC TGC ACA GTA GCG A - 3', CTDS-f 5'-GCT GGG AGG CAA AGG CTA CAA-3' CTDS-r 5'-TCC TGC TCT GGG ACT CTC CT-3', TFF1-f 5'-CCC TGG TGC TTC TAT CCT AAT A-3', TFF1-r 5'-AGA AGC GTG TCT GAG GTG TCC-3', GAPDH-f 5-GAA GGT GAA GGT CGG AGT-3', GAPDH-r 5-CAT GGG TGG AAT CAT ATT GGA-3'. Quantitative Real Time PCR (qRT-PCR) was performed with SYBR Green PCR Master Mix (Roche Diagnostic, Basel, Switzerland) in a LightCycler® 480 Real-Time PCR System instrument (Roche Diagnostic, Basel, Switzerland). Samples were run in triplicates and the efficiency of each primer was calculated utilizing an internal standard control [46]. All values were normalized for *GAPDH*.

### Co-immunoprecipitation assay

Sub-confluent MCF7 cells were harvested and proteins were prepared as follows: the cell pellet was resuspended in lysis buffer (20 mM Tris HCl pH 8, 137 mM NaCl, 10% glycerol, 1% NP40, 2 mM EDTA). 3 mg of proteins was immunoprecipitated, utilizing 4 μg of ERα antibody or mouse IgG overnight at 4°C. Extracts were incubated with antibodies and protein A/G beads (Pierce) for 3 h at 4°C. Immunopellets were washed extensively and subjected to SDS-PAGE followed by immunoblot analyses to detect pRb and ERα proteins.

### Immunofluorescence

MCF7 scramble and pRb KD cells were seeded on cover slips and grown in hormone-free medium for three days. Cells were treated with 17-β-estradiol or ethanol (as a negative control) for 45 minutes and fixed in 4% paraformaldehyde, permeabilized with phosphate-buffered saline (PBS) containing 1% Triton X-100 and 1% bovine serum albumin, and blocked in blocking buffer (PBS containing 8% bovine serum albumin). Cells were then incubated with total ERα antibody diluted at 1:100 (HC-20). After three washing with PBS, Alexa Fluor dyes (Invitrogen) were applied in blocking buffer as a secondary antibody. Nuclei were stained with 2',6'-diamidino-2-phenylindole (DAPI) in an antifade mounting medium (Vector laboratories, Burlingame, Calif.).

### Mice

All experiments with mice were approved by Temple IACUC committee. pRb floxed (FVB;129) and WAP-CRE (B6.Cg) mice were obtained from MMHCC (Mouse Models of Human Cancers Consortium). Mammary tissue was obtained from age-, parous- matched female mice, one day post-partum. Tissues were homogenized in Lysis Buffer (20 mM Tris HCl pH 8, 137 mM NaCl, 10% glycerol, 1% NP40, 2 mM EDTA) and 5-10 ug of protein extract was analysed by western blot.

### Statistical analysis

Statistical analysis was performed using GraphPad software by applying Student's t-test.

## SUPPLEMENTARY FIGURES


