# Supplementary material for: pRb controls Estrogen Receptor alpha protein stability and activity
Source: Oncotarget. 2013 Jun 3;4(6):875–83. doi: 10.18632/oncotarget.1036 (PMC3757244; doi:10.18632/oncotarget.1036)
Supplement: Supplementary file 1 [file oncotarget-04-875-s001.pdf]

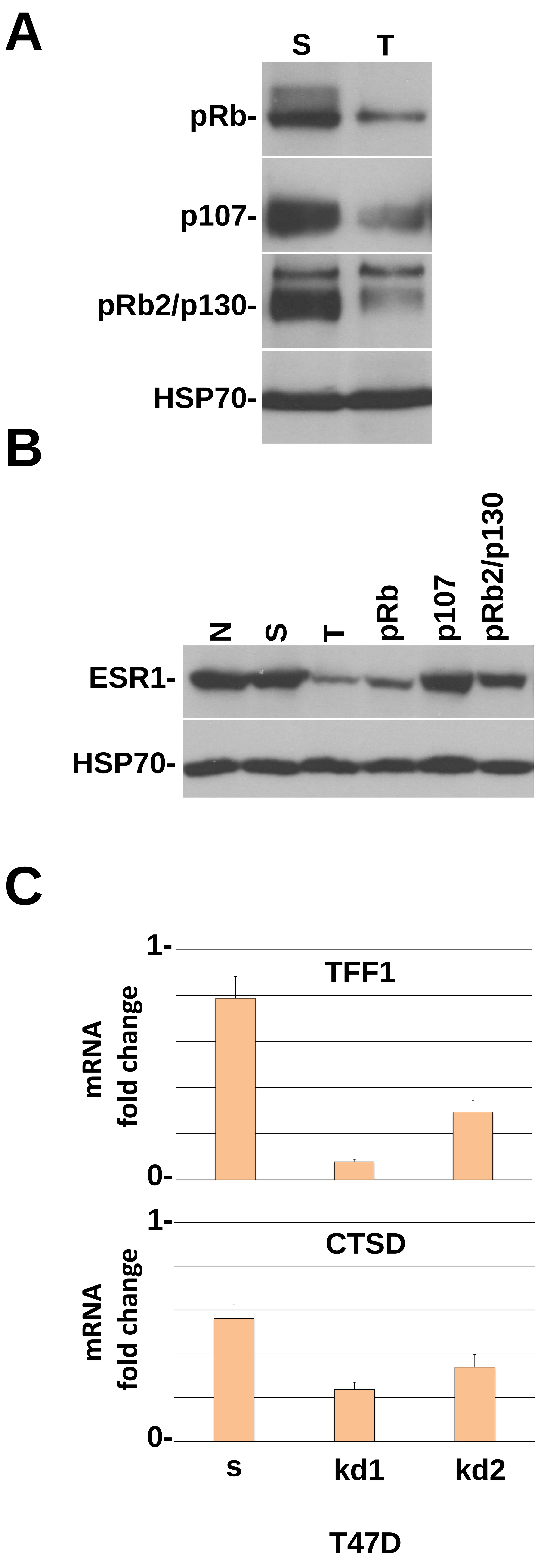

Supplemental Figure 1: (**A**) Testing the kd efficiency of *pRb*, *p107* and *pRb2/p130*. (**B**) Analysis of *pRb*, *p107* and *pRb2/p130* MCF7 kd cells. Only the loss of *RB1* down regulates the expression of the ESR1 protein. HSP70 was utilized as a loading control. T, triple knock down cells. (**C**) Loss of *RB1* down regulates the expression of *TFF1* and *CTSD*, two target genes of ESR1.

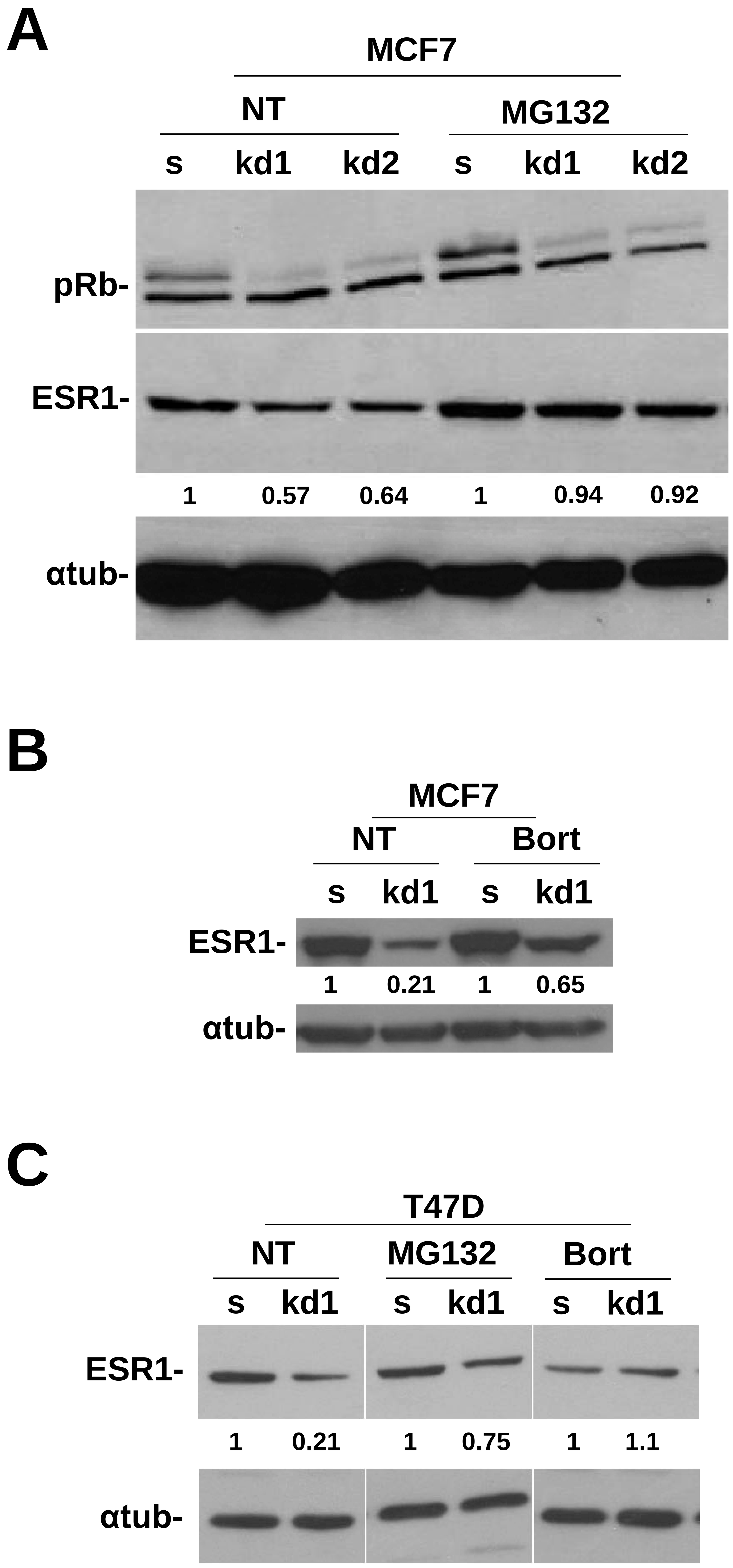

Supplemental Figure 2: (**A**) MG132 treatment recovered the expression of ESR1 in *RB1* kd1 and kd2 MCF7 cells. (**B**) Bortezomib provides the same results as MG132 in *RB1* kd1 cells. (**C**) MG132 and Bortezomib rescue the expression of ESR1 in T47D *RB1* kd1cells.
